# Incorporating Scannable Forms into Immunization Data Collection Processes: A Mixed-Methods Study

**DOI:** 10.1371/journal.pone.0049627

**Published:** 2012-12-18

**Authors:** Christine L. Heidebrecht, Susan Quach, Jennifer A. Pereira, Sherman D. Quan, Faron Kolbe, Michael Finkelstein, David L. Buckeridge, Jeffrey C. Kwong, Public Health Agency of Canada/Canadian Institutes of Health Research Influenza Research Network (PCIRN) Vaccine Coverage Theme Group

**Affiliations:** 1 Infectious Diseases, Public Health Ontario, Toronto, Ontario, Canada; 2 Centre for Innovation in Complex Care, University Health Network, Toronto, Ontario, Canada; 3 Communicable Disease Control Directorate, Toronto Public Health, Toronto, Ontario, Canada; 4 Dalla Lana School of Public Health, University of Toronto, Toronto, Ontario, Canada; 5 Department of Epidemiology, Biostatistics and Occupational Health, McGill University, Montreal, Québec, Canada; 6 Direction de santé publique, Agence de la santé et des services sociaux de Montréal, Montréal, Québec, Canada; 7 Institute for Clinical Evaluative Sciences, Toronto, Ontario, Canada; 8 Department of Family and Community Medicine, University of Toronto, Toronto, Ontario, Canada; University of British Columbia, Canada

## Abstract

**Introduction:**

Individual-level immunization data captured electronically can facilitate evidence-based decision-making and planning. Populating individual-level records through manual data entry is time-consuming. An alternative is to use scannable forms, completed at the point of vaccination and subsequently scanned and exported to a database or registry. To explore the suitability of this approach for collecting immunization data, we conducted a feasibility study in two settings in Ontario, Canada.

**Methods and Findings:**

Prior to the 2011–2012 influenza vaccination campaign, we developed a scannable form template and a corresponding database that captured required demographic and clinical data elements. We examined efficiency, data quality, and usability through time observations, record audits, staff interviews, and client surveys. The mean time required to scan and verify forms (62.3 s) was significantly shorter than manual data entry (69.5 s) in one organization, whereas there was no difference (36.6 s vs. 35.4 s) in a second organization. Record audits revealed no differences in data quality between records populated by scanning versus manual data entry. Data processing personnel and immunized clients found the processes involved to be straightforward, while nurses and managers had mixed perceptions regarding the ease and merit of using scannable forms. Printing quality and other factors rendered some forms unscannable, necessitating manual entry.

**Conclusions:**

Scannable forms can facilitate efficient data entry, but certain features of the forms, as well as the workflow and infrastructure into which they are incorporated, should be evaluated and adapted if scannable forms are to be a meaningful alternative to manual data entry.

## Introduction

Individual-level, electronically captured data are ideal for obtaining and tracking comprehensive immunization coverage information, and for facilitating rapid, evidence-based decision-making and planning [1]. Several jurisdictions across Canada use this approach [2], but in circumstances where electronic data capture at the point of vaccination is not feasible, initial capture on paper forms followed by manual data entry is required to populate electronic immunization information systems (IIS), a resource-intensive process [3].

One alternative is to use scannable forms, which facilitate automated data transfer from paper forms to electronic databases or registries. This approach to data collection has been applied in various clinical and research settings [4]–[10], and we hypothesized that it would be more efficient and accurate than manual data entry [11]–[13].

To explore the suitability of this approach for collecting influenza immunization data in diverse settings, and to obtain data to inform larger studies and guide broader-scale implementation, we conducted a feasibility study in two settings in Ontario, Canada during the 2011–2012 influenza immunization campaign.

## Methods

### Ethics Statement

We obtained ethics approval from the University of Toronto’s Health Sciences Research Ethics Board and the Research Ethics Committee of the Halton Region Health Department (HRHD). Participation in the study was contingent on agreement with terms and conditions specifying that each organization was responsible for data management and for remaining compliant with jurisdictional privacy legislation. All staff participants were asked to provide written informed consent before data collection began. We provided prospective survey respondents with an information sheet together with the survey, and assumed that by returning a completed questionnaire, a client was providing consent. All data were analyzed anonymously.

We identified and approached prospective participant organizations based on knowledge about current data processing practices, and a convenience sample of interested organizations was chosen to participate. These included HRHD, a local public health department serving the general population, and Rockwood Terrace (RT), a continuing care organization in Grey Bruce region administering influenza vaccines to employees. (Rockwood Terrace also administers vaccinations to its residents, but resident immunizations forms were not included in this pilot work.) We assessed the feasibility of scannable forms by examining data quality, efficiency, and usability.

We first explored the usability, data quality, and cost of several scanning solutions. Based on this assessment, we selected AutoData® Scannable Office as the most appropriate software for this feasibility study, and we used this software in conjunction with a desktop scanner with auto-feed and duplex scanning capabilities to populate a database.

### Data Flow

**Figure 1:**
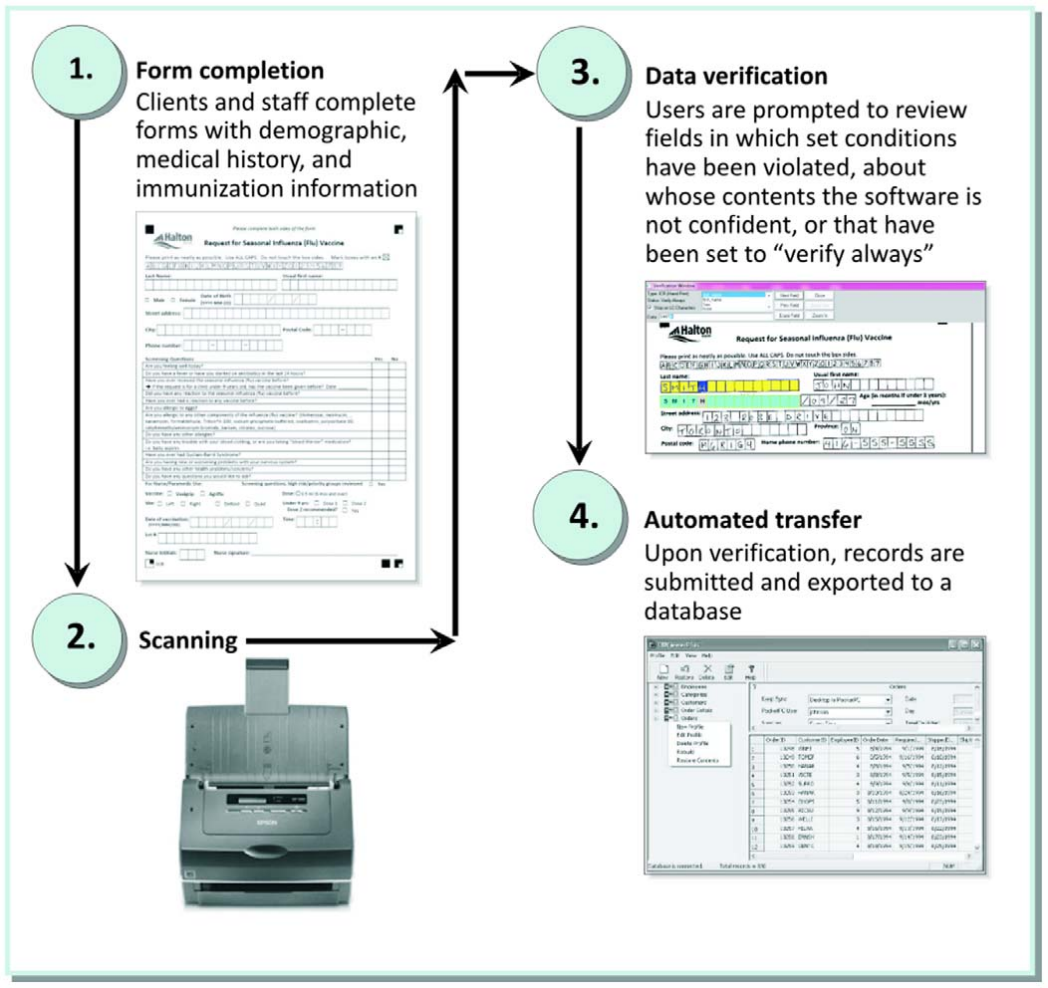
Data Flow.

Figure 1 illustrates data flow from form completion to electronic data capture. Scannable immunization consent forms were completed at the point of vaccination. In HRHD, clients completed demographic, medical history, and consent fields, while clinic staff recorded immunization information. At RT, the vaccinating nurse completed all fields. Forms were subsequently scanned and verified by each organization to ensure the accuracy of the scanned data. During verification, users were presented with content as interpreted by the software as well as an image of the original completed form. Users were prompted to review fields where set conditions were violated, where the software was not confident about the content of the field, or where the field was set to require validation. Content interpreted inaccurately by the software could be corrected by the user. Data were then transferred to a database to which the form’s field structure had been designed to correspond. Scannable Office can transfer scanned data directly to a new or pre-existing Microsoft® Access database or Excel spreadsheet, or any Open Database Connectivity (ODBC) compliant database.

### Implementation

#### Form design

We worked with each participating organization to develop a scannable paper form that captured the same demographic and clinical information collected routinely during influenza immunization campaigns. Hand-printed letters or numbers had to be written in individual boxes to be recognized and interpreted by the optical and intelligent character recognition features of the software; forms were designed to approximate the appearance of traditional forms while incorporating these boxes. Scannable elements consisted of hand-print fields and tick-box fields, to which a range of verification and quality assurance features were assigned.

▪ All hand-print fields were set to “verify always”
▪ Restrictions were set on the type (alphabetic, numeric, or both) and pattern of characters that were permissible in certain hand-print fields (e.g. postal code)
▪ Tick-box responses were restricted to one per question
▪ As appropriate, tick-box fields were set to “verify if no selection”

Scannable barcodes, each reflecting a different number, were incorporated into HRHD’s forms so that each client’s form would be assigned a unique identifier; the barcode field in the database was programmed to prevent more than one of the same barcode number from being entered. This was done to ensure that each electronic record could be traced back to its respective paper form, and to prevent duplicate records from being created.

Forms for both participating organizations comprised a single, double-sided page, and included instructions (and examples) for completion: print neatly, capitalize all letters, keep all numbers and letters within the lines of boxes, and use X’s to mark tick-boxes.

A unique identifier and corresponding scannable locator symbols – facilitating recognition of the form’s field structure – were automatically assigned to each form during design.

#### Database development

The data needs of each organization dictated the type of database to which data from each form would be transferred. RT chose to use an Access database to store data. Access was identified as the target repository when the first form was scanned, and the software automatically mapped the fields developed during form design to the database in this platform. This database was populated after each form had been scanned and verified.

HRHD, anticipating a much larger dataset and needing to incorporate more complex data management and quality assurance elements, selected a Microsoft SQL Server (MSSQL) database. As above, a table reflecting the form’s properties was automatically created in Access, after which the structure was imported into the MSSQL database. In order for each form field to be correctly associated with its respective field in this database, HRHD personnel were required to manually map fields using Scannable Office’s Data Mapper. Again, once this structure had been established, data from each form were transferred automatically upon verification.

### Data Collection and Analysis

#### Efficiency

We observed staff scanning and verifying a sample of all completed forms; the time (in seconds) required for each form to be scanned and verified was recorded by a member of the research team (CLH). As a comparison, we also recorded the time required to manually enter data into the same database. The same investigator conducted observations in each organization; the total time spent observing staff ranged from three to ten hours. We compared mean data entry times for the two approaches using t-tests.

Insufficient variability data for these data entry approaches meant that we were unable to perform a valid sample size calculation. As this was an exploratory study, we used a feasibility approach to sample size determination, anticipating that our findings would be able to facilitate robust calculations in the future. At RT the number of observations was based on the number of forms completed overall, and at HRHD we worked with program staff to identify the number of forms that they could reasonably process without compromising routine duties.

#### Data quality

Staff at participating organizations conducted record audits of both scanned and manually entered forms, documenting all occurrences of discordance between selected data elements recorded on paper forms and in corresponding electronic records. Data elements were selected based on published recommendations [14] and participant organization priorities. We determined rates of agreement for each field as well as overall error rates by type of field (hand-print, tick-box), and compared error rates between scanned and manually entered records by estimating binomial exact confidence intervals.

#### Usability

We conducted semi-structured in-person and telephone interviews with scanning personnel, database developers, program managers, and nursing staff at both organizations, exploring users’ perceptions of implementation steps, ease of use, data quality, and usefulness of data. Interviews were conducted with all individuals in these roles who interacted with the system, with the exception of vaccine administrators at HRHD, of which there were many; we determined that a sample size of four such staff would provide us with an appropriate range of perceptions regarding staff form completion. (At RT, a single staff member was responsible for administering vaccines for the duration of this pilot.) All staff who were invited to participate agreed to be interviewed. The same team member who timed data entry conducted the staff interviews. With participants’ consent, we recorded and transcribed all interviews. We performed thematic content analysis by assigning codes to concepts identified in each transcript and then categorized and sorted these coded concepts, facilitating the emergence of key themes.

HRHD clinic staff approached a convenience sample of clients during the 15-minute post-vaccination waiting period and asked them to complete a short questionnaire about their form completion experience (Appendix). (At RT the vaccinating nurse completed each vaccinnee’s form in its entirety so it was not necessary to seek feedback from clients.) We examined the frequency of quantitative responses and conducted chi-square tests to explore associations between perceptions and demographic characteristics. Open-ended responses were grouped into common themes.

In addition, we administered short questionnaires to charge nurses and clinic facilitators at community clinics at the same organization. We asked staff whether or not they had been approached by clients with questions about the form, and if so, to describe the nature and frequency of their concerns. We recorded responses on an observation form and analyzed these alongside interview data.

We performed statistical analyses using StataCorp Stata® 10, and ran qualitative queries in QSR NVivo® 8.

## Results

During the 2011–2012 influenza immunization campaign, all immunizations administered at HRHD public clinics and all staff immunizations and declinations at RT were documented on scannable forms.

### Efficiency

The time required to scan and verify forms at HRHD was significantly shorter than manual data entry, whereas at RT there was no difference (Table 1). Scannable Office has a batch scan setting that allows several forms to be scanned consecutively before each individual form is verified, leaving users free to engage in other tasks. Neither organization used this feature, but when we excluded the time required to scan each form and compared verification-only to manual data capture, the processing time for scanned forms was significantly faster than manually entered forms for both organizations.

**Table 1:** Data capture: mean time (in seconds) per immunization record.

Organization	*n*	Mean	Mean difference (95% CI)	p-value
**Halton Region Health Department**				
Manual data capture	201	69.5		
Scanning + verification	202	62.3	−7.3 (−10.0, −4.6)	<0.01
Verification only	191	56.9	−12.6 (−15.3, −9.9)	<0.01
**Rockwood Terrace** †				
Manual data capture	47	35.4		
Scanning + verification	43	36.6	1.2 (−2.8, 5.3)	0.55
Verification only	43	26.5	−8.9 (−12.9, −4.9)	<0.01

† The number of forms examined in this organization is reflective of both a smaller population and the fact that several forms’ locator symbols had been skewed during printing, rendering those pages unscannable.

One RT staff member was responsible for scanning and verifying all forms, but at HRHD, multiple individuals performed these tasks. We observed two of these staff members, and when we analyzed time by user we found no difference in mean time for scanning and verification, but did detect a difference between users’ manual data entry time (mean difference: 3.9 s (95% CI: 1.0, 6.9)).

### Data Quality

Record audits revealed high levels of agreement between paper forms and their respective electronic records for both scanned and manually entered data (Table 2). The individual data fields reported in Table 2 were selected primarily based on clinical importance, although we limited our reporting of HRHD’s high-risk/priority tick-box fields to a subset whose likelihood of error we deemed to be equivalent (over 65 years, provider of essential services). The number of discordant pairs observed in some fields in the scanned group was slightly higher compared to those that were manually entered, although these differences were not statistically significant.

**Table 2:** Agreement between paper forms and electronic records by data entry approach.

			Halton Region Health Department	Rockwood Terrace
Data element			# of discordant pairs (% disagreement)	# of discordant pairs (% disagreement)
			(n = 200 scanned; 200 manually entered)	(n = 47 scanned; 47 manually entered)
Surname				
		Scanned	6 (3.0)	3 (6.4)
		Manually entered	6 (3.0)	1 (2.1)
Date of birth				
		Scanned	7 (3.5)	Not collected
		Manually entered	3 (1.5)	
Postal code				
		Scanned	12 (6.0)	Not collected
		Manually entered	4 (2.0)	
Date of vaccination				
		Scanned	2 (1.0)	0
		Manually entered	0	0
Lot #				
		Scanned	3 (1.5)	3 (6.4)
		Manually entered	2 (1.0)	0
Consent to vaccination				
		Scanned	Not examined†	1 (2.1)
		Manually entered		0
Staff department				
		Scanned	Not collected	1 (2.1)
		Manually entered		0
Patient contact category				
		Scanned	Not collected	1 (2.1)
		Manually entered		0
Over 65 years				
		Scanned	0	Not collected
		Manually entered	0	
Provider of essential services				
		Scanned	0	Not collected
		Manually entered	0	
**Error rates by type of data element** ‡				
**Handprint fields**			**Total errors**	**Total errors**
			(%; 1600 data fields)	(%; 188 data fields)
	Scanned		48 (3.0)	7 (3.7)
	Manually entered		26 (1.6)	1 (0.5)
**Tick-box fields**			**Total errors**	**Total errors**
			(%; 1600 data fields)	(%; 141 data fields)
	Scanned		0	3 (2.1)
	Manually entered		4 (0.25)	0

† Consent to vaccination is captured on HRHD forms, but because the entire database consists of vaccinated individuals – in contrast to Rockwood Terrace, whose database reflects both immunized and non-immunized individuals – we did not examine this data element.

‡ Includes elements not reported individually above because they were perceived to be of lower clinical importance.

When we grouped all audited data elements into hand-print and tick-box fields, we found higher disagreement in handprint fields when scanned compared to when manually entered in both organizations, although these differences were not significant. We also observed non-significant increases in discordance in scanned tick-box fields for RT and in manually entered tick-box fields for HRHD.

We considered the possibility that some records containing multiple errors were responsible for a greater proportion of overall errors, but found that of the 494 records audited, six scanned and three manually entered forms each contained two errors. All remaining records contained one or no errors.

### Usability

We assessed the usability of the scanning approach by examining the perceptions of users engaged in implementation, form completion, and scanning and verification roles. At HRHD, we interviewed one manager, two database personnel, four vaccine administrators, one staff member responsible for scanning/verification, and one individual who was involved in database design as well as scanning/verification, and administered questionnaires to two charge nurses, two clinic facilitators, and 198 clients. At RT, we conducted interviews with one manager and one staff member responsible for vaccine administration and scanning/verification.

The database personnel interviewed at HRHD were involved with form design and integrating the form’s field structure with the MSSQL database; one of these respondents was also involved with scanning. These individuals described the initial transfer of scanned data to Access as very straightforward, but indicated that creating an MSSQL database and appropriately mapping the scannable form’s field structure to this database required technical support from AutoData®. After initial difficulties had been overcome, however, database personnel were comfortable addressing any issues that arose.

Questionnaires were completed by 198 clients at HRHD clinics; the majority (88%) of respondents were 31 to 80 years of age, and 50% were female. Most clients reported that instructions were clear (81%), the experience of writing letters/numbers in individual boxes was the same or easier than other forms (88%), completing this type of form took the same amount of time or less than other types of forms (87%), and there were no parts of the form that they found confusing (84%). Chi-square tests did not reveal any statistically significant associations between responses to those questions and age or sex.

Some clients noted that these forms contained more space than others, and that the allocation of one box per character made the form easier to complete, while others commented that boxes were too small and staying inside the lines of each box was difficult. Notable recommendations included incorporating clearer instructions about completing tick-boxes with “X”s, allowing check marks to be used, increasing the character box size, and offering clipboards to improve ease of completion while waiting in line. While few clients approached clinic staff with questions about the scannable elements on the immunization consent forms, the observations that nurses and clinic facilitators made regarding areas of difficulty for clients corroborated these findings.

Nurses described varying form completion experiences; some indicated that the forms were easy to complete, including one who preferred the individual text boxes because they compelled her to write more neatly, while others found that the new format took some time to get used to, and said that it was sometimes hard to stay within the lines of each box.

All users who were involved with scanning and verifying forms found the procedures user-friendly, although some form processing difficulties were observed. In both organizations, the software was unable to recognize a small proportion of forms; sometimes this was the result of wrinkled or soiled pages, but in most cases it was not possible to determine why the error had occurred. When the first page of a form scanned correctly but the second page was unscannable, care had to be taken to ensure that the contents of the first page were not duplicated upon manual entry. Close to half of RT forms’ locator symbols had been skewed during printing, which necessitated manual entry of their contents (these were timed as manual entries, not scanned entries). Occasionally, multiple pages would be scanned at once which was problematic because it meant that the first page of one form and the second page of another were considered the same form; in these cases the entry would be cancelled, and the forms separated and rescanned.

The software’s initial interpretation of the content of the hand-printed fields often required correction at the point of verification. Some fields, including names and addresses, required more corrections than others during this phase, likely because of the length of the field, and in the case of address, because the field was not restricted to either alphabetic or numeric characters. Further, several barcodes on HRHD forms were not recognized and had to be manually entered. Commenting on the concentration that is required during verification, one individual who was involved with scanning highlighted that the quality of the data exported was “very reliant on the attention of the person who was authorizing it.”

When asked to compare their scanning and manual data entry experiences, users at RT described similar, positive perceptions of both approaches, while in HRHD scanning was perceived to be preferable, due to speed of data entry as well as usability.

### Continued Use

At the conclusion of this pilot, RT and HRHD had each established a comprehensive electronic dataset of vaccinees, and users in both organizations acknowledged that the new approach to data collection afforded greater ease of access to many data elements. However, each organization differed in their attitudes about continuing to employ a similar approach in the future, based on perceived usability of more detailed data. HRHD was able to access required data (aggregate counts of age, sex, and high risk status) through traditional means (paper forms), and some program staff did not feel that the expenditure of resources was worthwhile for the influenza immunization program, especially since the vaccine must be administered every year. Further, because they provide a small proportion of all influenza vaccinations administered in the region, HRHD recognized that the coverage data they were able to assess were not representative of the entire population, and therefore limited in value. However, scanning as a mechanism for populating immunization registries was perceived as potentially valuable for other immunization programs that are administered exclusively by public health, because the resulting dataset would reflect the entire vaccinated population.

In contrast, as an institution requiring information about the influenza immunization status of each staff member, RT expressed a desire to continue to capture data electronically, whether through scanning or direct manual entry. The electronic availability of these data allowed management to assess coverage by department and level of patient contact – valuable for monitoring uptake throughout each campaign as well as emergency planning in the event of an outbreak – and also facilitated rapid data sharing with other personnel.

RT management acknowledged that the cost of the software ($4,670 USD) would likely be prohibitive for their small facility if it were used exclusively for immunization information, but that it would be possible to explore sharing data processing tasks with other associated institutions, and/or to use the program for other data collection needs.

## Discussion

This small-scale pilot was an initial exploration into the feasibility of using scannable paper forms to electronically capture individual-level immunization records required for the assessment of vaccine coverage, program planning, and rapid follow-up in the event of vaccine safety or effectiveness concerns. To our knowledge, this is the first study to examine the accuracy, efficiency, and usability of scannable forms as an immunization data collection tool in mass influenza vaccination settings.

We found that it was feasible to use scannable immunization forms for data collection in two distinct influenza vaccination settings. Personnel in both organizations used the scanning application successfully to capture high quality immunization data at an accuracy level comparable to manual data entry. We also observed that scanning is associated with shorter data processing times, which, when considered in the context of hundreds or thousands of vaccinees, means that fewer resources are required to compile these high quality datasets.

While our observations suggest that scannable forms can expedite data capture processes, reflection of and possible adaption to information infrastructure, clinic processes and workflow, and data needs will be required before this approach represents an ideal alternative to manual data entry for the creation of high quality electronic immunization records. As with any data collection process, organizations must consider how the data captured through scannable forms will be used, in order to ensure that the recipient database is developed appropriately. The desired sophistication of the database, including security measures, audit capabilities, anticipated queries and reports, and quality assurance mechanisms, will impact the level of technical expertise in database design required.

We observed variation in data quality and efficiency between organizations, system users, and individual forms. Both outcomes depend on user experience and concentration, and may have been influenced by the researcher’s presence during observation sessions. Several factors related to form content can also impact these outcomes. The software’s ability to recognize the content of hand-print fields is highly dependent on handwriting properties; irregularly-shaped characters, lower-case letters, and printing that touches the sides of character boxes can all compromise the software’s interpretation. Reviewing and correcting these fields is thus a critical but potentially time-consuming process. Further, printing quality can impact overall scannability, and unscannable forms must be manually entered. Our recommendations to improve data quality and reduce processing time are outlined in Table 3.

**Table 3:** Recommendations to Enhance Performance.

• Set all hand-print fields to “verify always”.
•Reduce the number of hand-print fields, for example by including tick-boxes reflecting age or postal code ranges, and pre-printing vaccination dates and lot numbers.
• Eliminate fields in which both alphabetic and numeric characters are permitted (e.g. address), unless a consistent character pattern can be identified and attributed (e.g. postal code); separate address into street number, street, and apartment/unit number fields.
• Increase the size of boxes in which letters and numbers are hand-printed.
• As others have suggested [11], explain to individuals who will engage in form completion that it is important that instructions are followed meticulously because the data will be scanned; in this pilot, nurses had been informed about the nature of the forms, but clients had not been explicitly told.
• Consider which data will be used in the future, to determine which fields need to be scanned. There may be elements of an immunization form that are critical for client and clinician decision-making, but which will not be analyzed later and therefore do not need to be scanned to a database.
• Utilize the batch scan setting, allowing numerous forms to be scanned in succession in advance of verification.
• Test the scannability of forms and adjust print-settings as necessary; once the quality of the printing is deemed acceptable, maintain consistent settings.
• Include appropriately-placed pictorial examples. Our request that X’s be used to mark tick-boxes created confusion for several clients, as well as some staff members. This instruction, which was intended to reduce the possibility that long check-marks could extend into, and erroneously mark, blank tick-boxes above, may have been more consistently followed had we included examples in closer proximity to the tick-box questions rather than at the beginning of the form.

We observed varying perceptions about the utility of the immunization database created during the course of this study. As a key informant at HRHD highlighted, there may be limited use for a dataset that reflects a small proportion of the total vaccinations administered in a population. However, it is important to consider that having these data available electronically may facilitate data sharing between vaccine providers and consequently support the assembly of more comprehensive immunization datasets. Panorama, an electronic public health surveillance system that is currently being implemented in several Canadian jurisdictions, includes immunization registry capabilities; scannable forms and character recognition software may serve as a valuable mechanism for populating this information system in areas where immunization data are not collected electronically at the point of care.

There are some limitations of this work. Because this was a pilot study, we limited the number of sites in which this system was implemented, and the number of observed data points. Therefore our findings may not represent the broader landscape of organizations who could find this approach to data capture useful. This pilot was limited to settings administering influenza vaccines; it would be valuable for future research on scannable forms to include other immunizations. The software used in this pilot study is one of many available applications that automate the transfer of data from paper to electronic format; while Scannable Office was deemed most appropriate for our study, other solutions offer additional features such as custom dictionaries, fax-to-scan functionality, and record audit capabilities, and are therefore worth exploring. Finally, we did not conduct a cost/benefit analysis. Based on our observation of efficiency in HRHD, automated data transfer may represent a cost-savings over manual data entry, but there are many factors that influence implementation costs, and these should be examined in the context of larger studies before valid conclusions about cost-effectiveness can be drawn.

Many barriers have impeded the adoption of point-of-care immunization data capture systems [15], and few jurisdictions currently maintain population-wide immunization registries. To optimize the value of immunization data that are collected, alternative solutions that facilitate population of individual-level records, while offering greater efficiency than manual data entry, are needed. This pilot has demonstrated that while scannable forms can facilitate efficient data entry, certain features of the forms as well as the workflow and infrastructure into which they are incorporated will have to be re-evaluated and adapted in order for this approach to serve as a meaningful alternative to manual data entry.
